# Supplementary material for: The Fate of IgE Epitopes and Coeliac Toxic Motifs during Simulated Gastrointestinal Digestion of Pizza Base
Source: Foods. 2022 Jul 6;11(14):2000. doi: 10.3390/foods11142000 (PMC9318710; doi:10.3390/foods11142000)
Supplement: Supplementary file 1 [file foods-11-02000-s001.zip › Daly-Wang et al. Supplementary Materials ver 12.pdf]

Supplementary Materials

Figure S1. The molecular function of identified wheat proteins determined by gene ontology analysis. Panel A; G0, B; G60, C; G120, D; D0, E; D60 and F; D120.

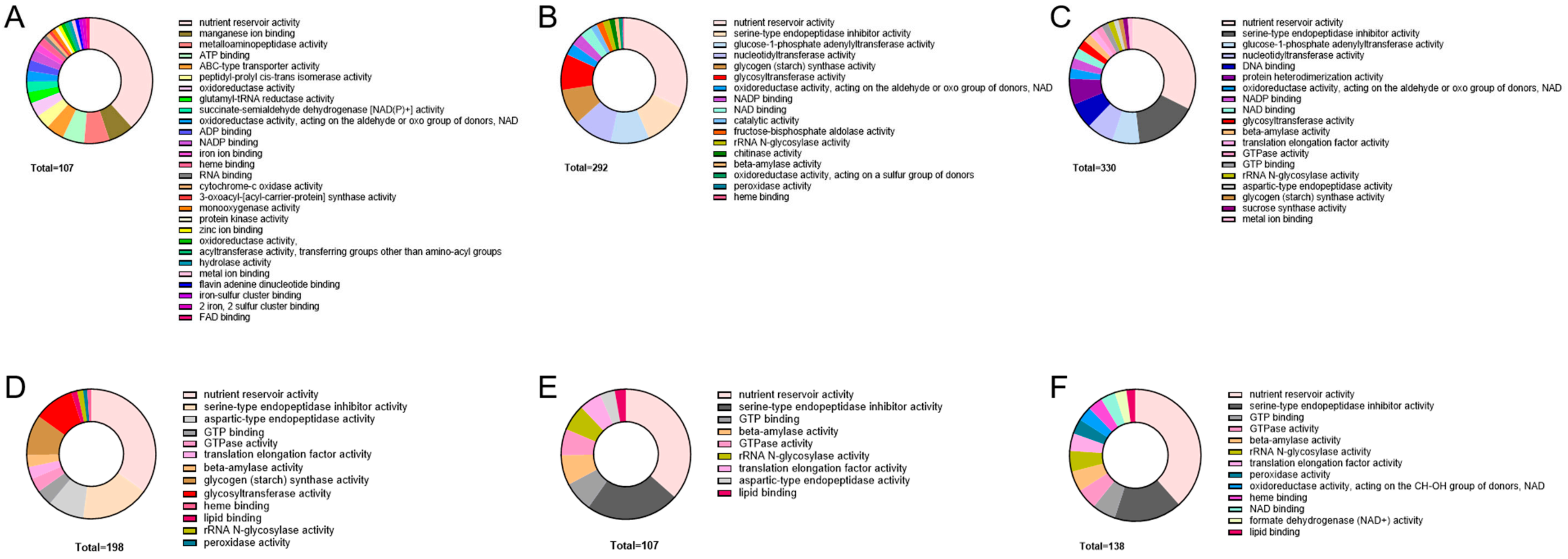

Figure S2. The molecular function of identified soy proteins determined by gene ontology analysis. Panel A; G0, B; G60, C; G120, D; D0, E; D60 and F; D120.

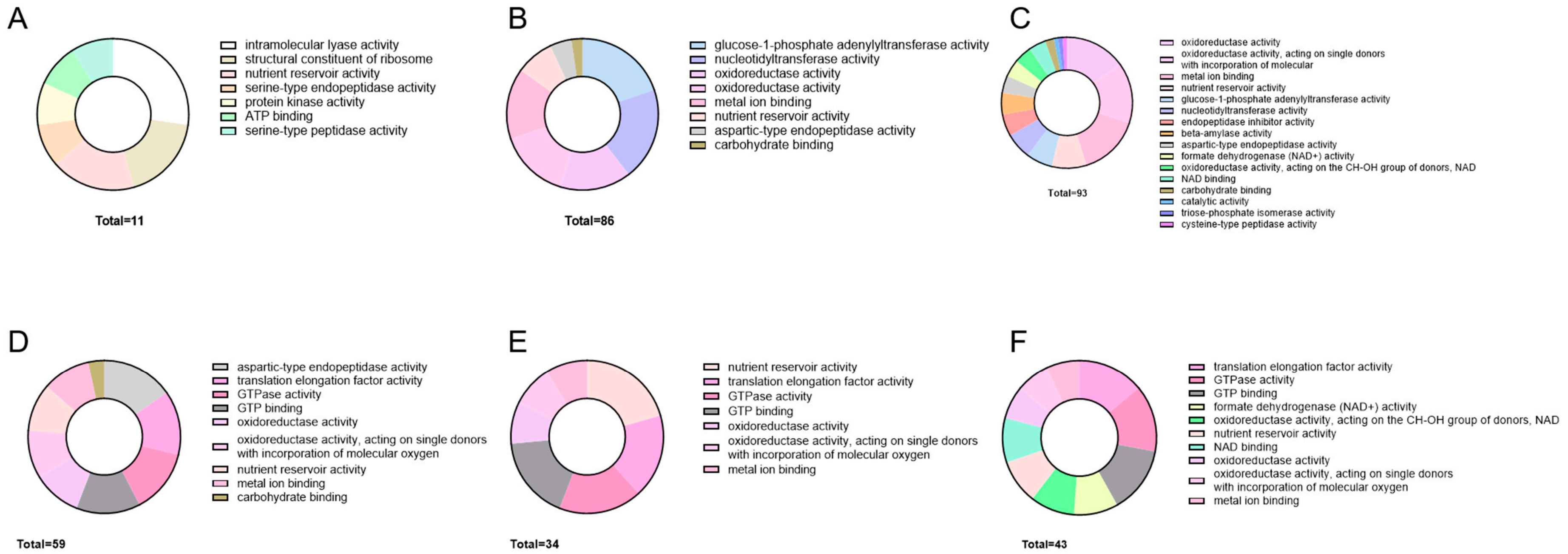

Figure S3. Peptide mapping of the LMW-GS allergen B2Y2Q7 at time points G120 (A) and D60 (B). Identified peptides mapped onto the parent protein B2Y2Q7 at time points G120 (A) and D60 (B). The sequenced bounded by a black box indicates the signal sequence, and those bounded in red and green represent IgE epitopes and coeliac toxic motifs, respectively. The repetitive domains with consensus repetitive motif PPFSQQQQ peptides are shown in blue boxes. Endoprotease cleavage sites are denoted by red dots. Blue indicates peptides with lower abundance according to spectral counts with gradient to red indicating increasing abundance.

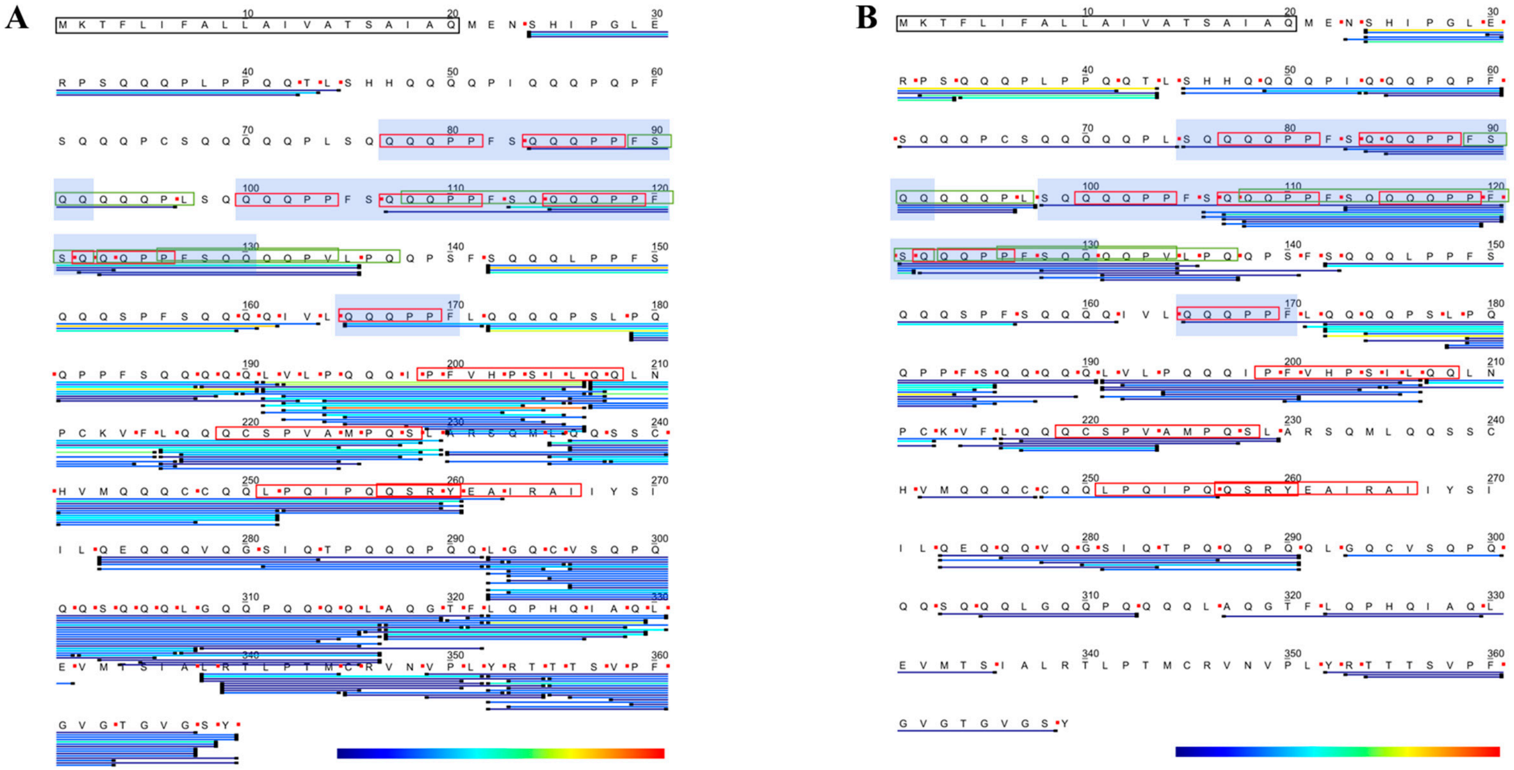

Figure S4. Predicted protease cleavage sites for the LMW-GS allergen B2Y2Q7 by pepsin (pH > 2) (A) and chymotrypsin (low specificity) (B).

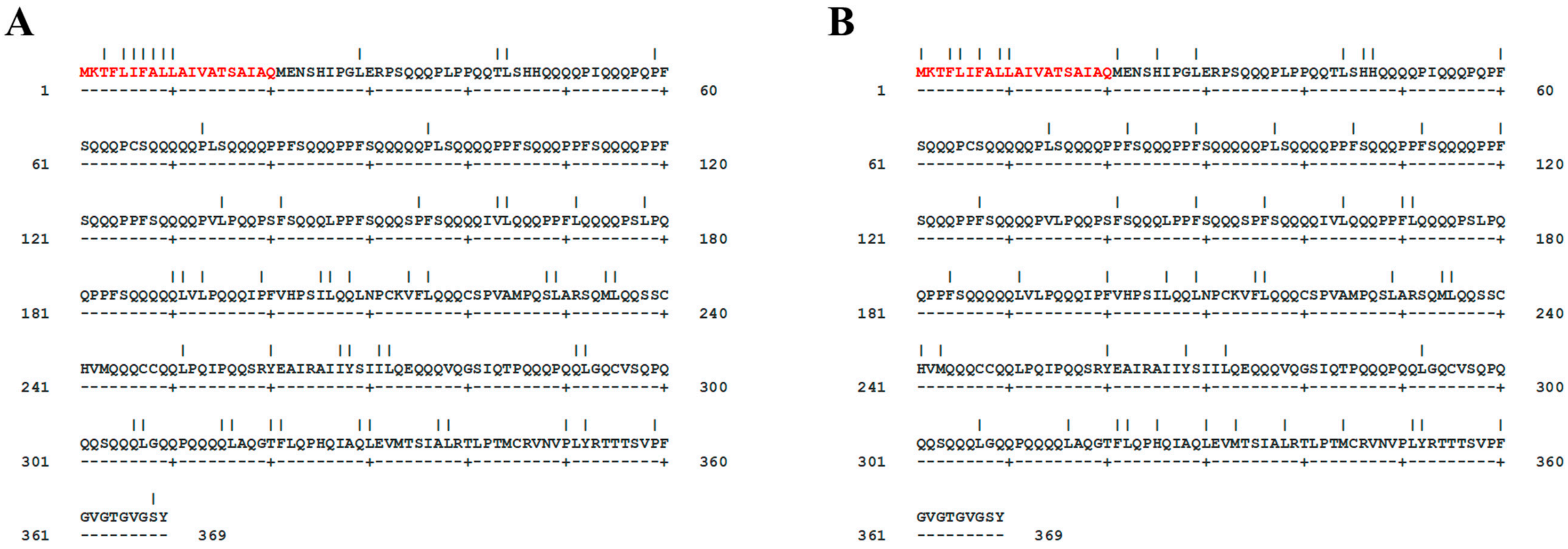

Figure S5. Nested overlapping peptide digestion products from the N-terminal repetitive domain 34-197 of LMW glutenin subunit B2Y2Q7.

34 40 50 60 70 80

QQQPLPP QQTLSHHQQQ QPIQQQPQPF SQQQPCSQQQ QQPLSQQQQP

SQQQQP

SQQQ QQPL

NESTED SET 4

90 100 110 120 130

PFSQQQPPFS QQQQQPLSQQ QQPPFSQQQP PFSQQQQPPF SQQQPPFSQQ

PFSQQQPPFS QQQ

NESTED SET 1

SQQ QQPPFSQQQP PFSQQQ

NESTED SET 2

SQQQQPPF SQQQPPFSQQ

NESTED SET 3

S QQQQQPL

NESTED SET 5

140 150 160 170 180

QQPVLPQQPS FSQQQLPPFS QQQSPFSQQQ QIVLQQQPPF LQQQQPSLPQ

Q

190 197

QPPFSQQQQQ LVLPQQQ

Table S1. The Allerwheat and Allersoy allergen database lists. NA: UniProt accession not available.

Protein sub-group	WHO/IUIS Allergen nomenclature	Primary route of exposure	Allergen isoform UniProt accession number	PFAM family
Alpha/beta gliadin	Tri a 21	Food	D2T2K3	Tryp_alpha_amyl
Gamma gliadin	Tri a 20	Food	A0A060N479	Gliadin
			Q9SYX8	Gliadin
Omega-5 gliadin	Tri a 19	Food	Q402I5	Domainless
Low molecular weight glutenin	Tri a 36	Food	B2Y2Q7	Gliadin
High molecular weight glutenin	Tri a 26	Food	P10388	Glutenin_HMW
			Q45R38	Glutenin_HMW
Monomeric alpha-amylase inhibitor 0.28	Tri a 15	Airway	D2TGC3	Tryp_alpha_amyl
Dimeric alpha-amylase inhibitor 0.19	Tri a 28	Airway	Q4W0V7	Tryp_alpha_amyl
Tetrameric alpha-amylase inhibitor CM1/CM2	Tri a 29	Airway	C7C4X0	Tryp_alpha_amyl
			D2TGC2	Tryp_alpha_amyl
Tetrameric alpha-amylase inhibitor CM3	Tri a 30	Airway	P17314	Tryp_alpha_amyl
Tetrameric alpha-amylase inhibitor CM17	Tri a 40	Airway	Q41540	Tryp_alpha_amyl
Serpin	Tri a 33	Airway	Q9ST57	Serpin
Serine protease inhibitor-like protein	Tri a 39	Airway	J7QW61	potato_inhibit
Non-specific lipid transfer protein 1	Tri a 14	Airway	D2T2K2	Tryp_alpha_amyl
Endosperm transfer cell specific PR60 precursor	Tri a 44	Airway	A0A0G3F720	Domainless
Beta-amylase	Tri a 17	Airway	P93594	Glyco_hydro_14
Triosephosphate-isomerase	Tri a 31	Airway	Q9FS79	TIM
Glyceraldehyde-3-phosphate-dehydrogenase	Tri a 34	Airway	C7C4X1	Gp_dh_C<br>Gp_dh_N
Profilin	Tri a 12	Food	P49232	Profilin
			P49233	Profilin
			P49234	Profilin
			B6EF35	Profilin
Thioredoxin	Tri a 25	Airway	Q9LDX4	Thioredoxin
Thiol reductase homologue	Tri a 27	Airway	Q7Y1Z2	GILT
1-cys-peroxiredoxin	Tri a 32	Airway	Q6W8Q2	1-cysPrx_C<br>AhpC-TSA
Agglutinin isolectin 1	Tri a 18	Airway	P10968	Chitin_bind_1
Dehydrin	Tri a 35	Airway	D2TE72	Dehydrin
Alpha purothionin	Tri a 37	Food	Q9T0P1	Thionin
Mitochondrial ubiquitin ligase activator of NFKB 1	Tri a 41	Airway	A0A0G3F2P1	GIDE
Elongation factor 1 (EIF1)	Tri a 45	Airway	A0A0G3F715	Elf1
Hypothetical protein from cDNA	Tri a 42	Airway	A0A0G3F2F5	Domainless
Hypothetical protein from cDNA	Tri a 43	Airway	A0A0G3F5F7	SCAI
Hydrophobic protein from soybean	Gly m 1	Food	Q9S8F3	Hydrophob_seed
			Q9S8F2	
Defensin	Gly m 2	Airway	NA	NA

Protein sub-group	WHO/IUIS Allergen nomenclature	Primary route of exposure	Allergen isoform UniProt accession number	PFAM family
Profilin	Gly m 3	Food	O65809	Profilin
			O65810	Profilin
Pathogenesis-related protein, PR-10, Bet v 1 family member	Gly m 4	Food	P26987	Bet_v_1
Beta-conglycinin (vicilin, 7S globulin)	Gly m 5	Food	O22120	Cupin_1
			Q9FZP9	Cupin_1
			P25974	Cupin_1
Glycinin (legumin, 11S globulin)	Gly m 6	Food	P04776	Cupin_1
			P04405	Cupin_1
			P11828	Cupin_1
			Q9SB11	Cupin_1
Seed biotinylated protein	Gly m 7	Food	C6K8D1	Domainless
2S albumin	Gly m 8	Food	P19594	Tryp_alpha_amyl

Table S2. Peptides mapped to total wheat and soy proteins, and the LMW-GS allergen B2Y2Q7 that were found consistently through timepoints throughout simulated digestion. 'n.a.' not applicable.

**Wheat**						
	**Timepoints**					
	**G0**	**G60**	**G120**	**D0**	**D60**	**D120**
**Common from previous timepoint**	n/a	46	1333	237	693	976
**Common from G0**	n/a	46	41	13	9	7
**Total identified**	165	1716	2677	2373	2314	1664
**Soy**						
	**Timepoints**					
	**G0**	**G60**	**G120**	**D0**	**D60**	**D120**
**Common from previous timepoint**	n/a	17	214	23	53	72
**Common from G0**	n/a	17	12	3	1	0
**Total identified**	35	273	474	150	110	119
**B2Y2Q7**						
	**Timepoints**					
	**G0**	**G60**	**G120**	**D0**	**D60**	**D120**
**Common from previous timepoint**	n/a	n/a	90	35	57	65
**Common from G60**	n/a	n/a	90	28	14	9
**Total identified**	0	110	151	164	104	100

Table S3. Wheat allergens identified in each digestion timepoint with at least one unique peptide. Highlighted orange box indicates that UniProt accession was found in that timepoint.

	Timepoint					
	G0	G60	G120	D0	D60	D120
**Tri a 12**	P49234	P49234	P49234	P49234	P49234	P49234
	B6EF35	B6EF35	B6EF35	B6EF35	B6EF35	B6EF35
	P49233	P49233	P49233	P49233	P49233	P49233
	P49232	P49232	P49232	P49232	P49232	P49232
**Tri a 14**	D2T2K2	D2T2K2	D2T2K2	D2T2K2	D2T2K2	D2T2K2
**Tri a 15**	D2TGC3	D2TGC3	D2TGC3	D2TGC3	D2TGC3	D2TGC3
**Tri a 17**	P93594	P93594	P93594	P93594	P93594	P93594
**Tri a 18**	P10968	P10968	P10968	P10968	P10968	P10968
**Tri a 19**	Q402I5	Q402I5	Q402I5	Q402I5	Q402I5	Q402I5
**Tri a 20**	Q9SYX8	Q9SYX8	Q9SYX8	Q9SYX8	Q9SYX8	Q9SYX8
	A0A060N479	A0A060N479	A0A060N479	A0A060N479	A0A060N479	A0A060N479
**Tri a 21**	D2T2K3	D2T2K3	D2T2K3	D2T2K3	D2T2K3	D2T2K3
**Tri a 25**	Q9LDX4	Q9LDX4	Q9LDX4	Q9LDX4	Q9LDX4	Q9LDX4
**Tri a 26**	Q45R38	Q45R38	Q45R38	Q45R38	Q45R38	Q45R38
	P10388	P10388	P10388	P10388	P10388	P10388
**Tri a 27**	Q7Y1Z2	Q7Y1Z2	Q7Y1Z2	Q7Y1Z2	Q7Y1Z2	Q7Y1Z2
**Tri a 28**	Q4W0V7	Q4W0V7	Q4W0V7	Q4W0V7	Q4W0V7	Q4W0V7
**Tri a 29**	D2TGC2	D2TGC2	D2TGC2	D2TGC2	D2TGC2	D2TGC2
	C7C4X0	C7C4X0	C7C4X0	C7C4X0	C7C4X0	C7C4X0
**Tri a 30**	P17314	P17314	P17314	P17314	P17314	P17314
**Tri a 31**	Q9FS79	Q9FS79	Q9FS79	Q9FS79	Q9FS79	Q9FS79
**Tri a 32**	Q6W8Q2	Q6W8Q2	Q6W8Q2	Q6W8Q2	Q6W8Q2	Q6W8Q2
**Tri a 33**	Q9ST57	Q9ST57	Q9ST57	Q9ST57	Q9ST57	Q9ST57
**Tri a 34**	C7C4X1	C7C4X1	C7C4X1	C7C4X1	C7C4X1	C7C4X1
**Tri a 35**	D2TE72	D2TE72	D2TE72	D2TE72	D2TE72	D2TE72
**Tri a 36**	B2Y2Q7	B2Y2Q7	B2Y2Q7	B2Y2Q7	B2Y2Q7	B2Y2Q7
**Tri a 37**	Q9T0P1	Q9T0P1	Q9T0P1	Q9T0P1	Q9T0P1	Q9T0P1
**Tri a 39**	J7QW61	J7QW61	J7QW61	J7QW61	J7QW61	J7QW61
**Tri a 40**	Q41540	Q41540	Q41540	Q41540	Q41540	Q41540
**Tri a 41**	A0A0G3F2P1	A0A0G3F2P1	A0A0G3F2P1	A0A0G3F2P1	A0A0G3F2P1	A0A0G3F2P1
**Tri a 42**	A0A0G3F2F5	A0A0G3F2F5	A0A0G3F2F5	A0A0G3F2F5	A0A0G3F2F5	A0A0G3F2F5
**Tri a 43**	A0A0G3F5F7	A0A0G3F5F7	A0A0G3F5F7	A0A0G3F5F7	A0A0G3F5F7	A0A0G3F5F7
**Tri a 44**	A0A0G3F720	A0A0G3F720	A0A0G3F720	A0A0G3F720	A0A0G3F720	A0A0G3F720
**Tri a 45**	A0A0G3F715	A0A0G3F715	A0A0G3F715	A0A0G3F715	A0A0G3F715	A0A0G3F715

Table S4. Soy allergens identified in each digestion timepoint with at least one unique peptide
Highlighted orange box indicates that UniProt accession was found in that timepoint.

	Timepoint					
	**G0**	**G60**	**G120**	**D0**	**D60**	**D120**
**Gly m 1**	Q9S8F3	Q9S8F3	Q9S8F3	Q9S8F3	Q9S8F3	Q9S8F3
	Q9S8F2	Q9S8F2	Q9S8F2	Q9S8F2	Q9S8F2	Q9S8F2
**Gly m 3**	O65809	O65809	O65809	O65809	O65809	O65809
	O65810	O65810	O65810	O65810	O65810	O65810
**Gly m 4**	P26987	P26987	P26987	P26987	P26987	P26987
**Gly m 5**	O22120	O22120	O22120	O22120	O22120	O22120
	Q9FZP9	Q9FZP9	Q9FZP9	Q9FZP9	Q9FZP9	Q9FZP9
	P25974	P25974	P25974	P25974	P25974	P25974
**Gly m 6**	P04776	P04776	P04776	P04776	P04776	P04776
	P04405	P04405	P04405	P04405	P04405	P04405
	P11828	P11828	P11828	P11828	P11828	P11828
	Q9SB11	Q9SB11	Q9SB11	Q9SB11	Q9SB11	Q9SB11
**Gly m 7**	C6K8D1	C6K8D1	C6K8D1	C6K8D1	C6K8D1	C6K8D1
**Gly m 8**	P19594	P19594	P19594	P19594	P19594	P19594

Table S5. Frequency of N- and C-terminal side residues of alternative cleavage sites on LMW-GS allergen B2Y2Q7. The cleavages happened for repetitive peptides are excluded.

Residue	Frequency (N-/C-terminal side)				
	G60	G120	D0	D60	D120
**Q**	22/16	24/21	31/38	23/24	23/29
**S**	3/9	6/8	14/9	10/5	10/5
**P**	2/1	2/4	4/3	3/1	6/2
**C**	2/3	4/3	4/2	2/1	3/1
**G**	4/1	4/1	2/1	1/1	1/1
**V**	0/4	2/4	3/1	2/1	3/0
**E**	1/0	1/0	2/0	2/0	2/0
**A**	3/0	3/0	2/1	0/1	0/1
**N**	2/0	2/0	1/1	1/1	1/1
**F**	3/0	3/0	0/1	0/3	0/4
**R**	1/0	1/1	1/2	1/1	1/2
**I**	0/5	1/5	2/2	1/3	1/1
**M**	3/1	2/1	0/1	0/0	0/0
**T**	1/5	2/6	1/2	0/1	0/1
**H**	1/1	1/1	0/1	0/0	0/1
**L**	1/1	1/2	0/1	0/2	0/1
**Y**	0/2	0/2	0/0	0/0	0/0
**K**	1/0	1/0	1/1	1/1	1/1
**Total**	49/49	59/59	67/67	46/46	51/51
